# 西妥昔单抗联合塞来昔布对肺腺癌细胞KDR和AQP1表达的影响

**DOI:** 10.3779/j.issn.1009-3419.2013.12.02

**Published:** 2013-12-20

**Authors:** 洪刚 夏, 剑飞 叶, 宏宇 白, 长利 王

**Affiliations:** 1 300070 天津，天津医科大学研究生院 Graduate School of Tianjin Medical University, Tianjin 300070, China; 2 300280 天津，天津大港油田总医院胸外科 Department of Thoracic Surgery, Tianjin Dagang Oil Field General Hospital, Tianjin 300280, China; 3 300060 天津，天津市肿瘤医院胸外科 Department of Thoracic Surgery, Tianjin Tumour Hospital, Tianjin 300060, China

**Keywords:** 西妥昔单抗, 塞来昔布, 细胞凋亡, KDR, AQP1, Cetuximab, Celecoxib, Cell apoptosis, KDR, AQP1

## Abstract

**背景与目的:**

新辅助化疗是肺癌治疗的新进展，西妥昔单和合塞来昔布均是近年来新辅助化疗的常用药物，本研究旨在探讨表皮生长因子受体（epithelial growth factor receptor, EGFR）单克隆抗体西妥昔单抗（Cetuximab）联合环氧合酶2（cyclooxygenase-2, COX-2）抑制剂塞来昔布对肺腺癌A549细胞株凋亡和KDR、AQP1表达的影响。

**方法:**

A549细胞培养于RPMI-1640培养液中，实验分为正常对照组、西妥昔单抗1 nmol/L组、塞来昔布25 μmol/L组、西妥昔单抗1 nmol/L+塞来昔布25 μmol/L组。药物干预细胞48 h后，采用四甲基偶氮唑盐比色法测定细胞抑制率；采用流式细胞术测定细胞周期分布的变化；用Transwell小室法检测药物处理前后细胞侵袭力的变化。采用RT-PCR、Western blot检测*KDR*、*AQP1*基因和蛋白的表达情况。

**结果:**

西妥昔单抗和塞来昔布对A549细胞增殖有明显的抑制作用，两制剂联合作用时抑制作用更强（*P* < 0.01）。西妥昔单抗和塞来昔布均可诱导细胞凋亡，联合作用后细胞凋亡率更高（*P* < 0.01）；联合用药与单独用药相比，可使细胞发生明显的G_1_期阻滞（*P* < 0.01），进一步下调了*KDR*、*AQP1*基因和蛋白的表达（*P* < 0.05），细胞侵袭力明显降低。

**结论:**

西妥昔单抗和塞来昔布联合应用具有协同作用，可进一步抑制细胞的生长和迁移，下调了*KDR*、*AQP1*基因和蛋白的表达提示两药联合使用临床肺癌治疗可能具有很大的潜力。

## Abstract (en)

**Background and objective:**

Neoadjuvant chemotherapy is a new development in the treatment of lung cancer. In recent years, cetuximab and celecoxib have been commonly used in this procedure. This study aims to explore the effect of cetuximab combined with celecoxib on apoptosis and KDR and AQP1 expression in lung cancer A549 cells.

**Method:**

The cells were cultured in RPMI-1640 and then divided into four groups: control group, 1 nmol/L cetuximab group, 25 μmol/L celecoxib group, and 1 nmol/L cetuximab+25 μmol/L celecoxib group. The treatment time was 48 h. The mRNA and protein expression levels of KDR and AQP1 were detected by RT-PCR and Western blot, respectively. The apoptosis, proliferation, and invasive ability of A549 cells before and after transfection were examined using flow cytometry, MTT, and transwell methods.

**Results:**

Cetuximab and celecoxib inhibited the growth of A549 cells in a dose-dependent manner. Their combination produced a greater growth inhibition than when either was used alone (*P* < 0.01). Cetuximab and celecoxib both induced the apoptosis of A549 cells, and their combination produced a higher apoptosis rate (*P* < 0.01). Cetuximab in combination with celecoxib also induced G_1_ phase arrest and downregulated the expression of KDR and AQP1 in A549 cells (*P* < 0.05). As a result, the invasion ability of the A549 cells was significantly decreased.

**Conclusion:**

Cetuximab in combination with celecoxib can synergistically inhibit the growth of A549 cells and downregulate the expression of KDR and AQP1 in A549 cells. The combination of cetuximab and celecoxib is a potential strategy for lung cancer therapy.

肺癌是人类高发的恶性肿瘤，病死率在恶性肿瘤中居首位，其治疗失败和死亡的主要原因肺癌的侵袭和转移，新生血管的生成在肺癌的侵袭和转移中起着极其重要的作用^[1, 2]^。近年来研究^[3-6]^表明水通道蛋白1（aquaporins 1, AQP1）和血管内皮生长因子受体Ⅱ/激酶功能区受体（vascular endothelial growth factor Ⅱ, VEGFR2/kinase domain receptor, KDR）在许多肿瘤及其血管内皮细胞均有表达升高，在促进血管生成和肿瘤细胞增殖中起着关键作用。本研究以肺腺癌A549细胞株为研究对象，观察表皮生长因子受体（epithelial growth factor receptor, EGFR）单克隆抗体西妥昔单抗、环氧合酶-2（cyclooxygenase-2, COX-2）抑制剂塞来昔布单独及联用时对体外肺腺癌A549细胞增殖和凋亡及*KDR*、*AQP1*基因和蛋白表达的影响，探讨两者的协同关系及其机制，以期为临床治疗肺癌提供新思路和实验依据。

## 1 材料与方法

### 1.1 主要试剂和仪器

肺腺癌细胞株A549购自中国科学院上海细胞究所。RPMI-1640培养液、胎牛血清和胰蛋白酶均购自美国Gibco公司。SPB购自德国Merck公司，塞来昔布（Celecoxib，江苏恒瑞制药有限公司），西妥昔单抗（Cetuximab，德国默克公司），青霉素（100 U/mL）（北京化工厂），链霉素（100 U/mL）（Peprotech），5%四甲基偶氮唑蓝（MTT溶液，上海华美生物工程公司）；流式细胞仪（COULTER XL, Coulter）；550酶标仪（美国Biorad公司），生物倒置显微镜（Olympus CKX4）。

### 1.2 方法

#### 1.2.1 细胞培养和实验分组

肺腺癌细胞株A549购自中国科学院上海细胞研究所，培养在含10%胎牛血清的RPMI-1640培养液中，置饱和湿度5%CO_2_、37 ℃恒温培养箱中培养。细胞以1×10^6^个/100 mL培养瓶，常规培养24 h后如下分组进行药物处理，实验分组：对照组（PBS液1 mL处理组）；西妥昔单抗1 nmol/L组；塞来昔布25 μmol/L组；西妥昔单抗1 nmol/L+塞来昔布25 μmol/L组，药物浓度见参考文献^[7, 8]^。

#### 1.2.2 MTT比色实验

在96孔板上按5×10^4^个/孔接种A549细胞，如1.2分组，每组设三个平行重复孔，置于37 ℃、5%CO_2_的培养箱中培养48 h后，弃去培养液，生理盐水漂洗一遍，每孔加MTT 20 μL（浓度为5 mg/mL），放置孵箱内4 h后弃上清加入10%的SDS（十二烷基磺酸钠）200 μL，过夜。震荡15 min，用全自动酶标仪（美国Biorad公司）检测570 nm处的吸光度值（*A*值）。抑制率（%）=（*A*_对照组_-*A*_实验组_）/（*A*_对照组_-*A*_空白组_）×100%。

#### 1.2.3 流式细胞仪检测细胞周期

收集如1.2分组的不同处理组细胞，调整细胞浓度为1×10^6^个/L，冷PBS洗涤2次，加入70%的冷乙醇（4 ℃）固定24 h。洗涤细胞，与含10 μg/mL RNA酶的Tris-HCL缓冲液（pH7.4）共同孵育30 min。50 μg/mL碘化丙啶进行细胞DNA染色，1 h内通过流式细胞仪（COULTER XL, Coulter）；分析细胞DNA含量分布，计算出各个周期细胞的百分率。

#### 1.2.4 Transwell小室侵袭实验

在聚碳酸酯微孔滤膜上铺Matrigel 50 μg/孔，在聚合好的小室下室加入10%的胎牛血清做条件培养液，在上室加入按如1.2分组处理过的A549细胞悬液100 μL（细胞总数为3×10^5^个/L），置于培养箱中20 h后取出，用湿棉签仔细擦掉上室中未穿过的瘤细胞，95%的乙醇固定5 min，PBS轻轻漂洗3遍，进行HE染色，自然凉干，将上室的聚碳酸酯滤膜沿边缘用手术刀片小心取下，用树脂胶固定于玻片上（膜内面朝上），封片；高倍显微镜下记数穿膜的A549细胞数，每膜记数上、下、左、右、中5个视野的侵袭细胞数，计算平均值，每组设三个滤膜。

#### 1.2.5 RT-PCR检测*KDR*、*AQP1*基因表达

将各组处理后的细胞悬液分别经离心半径16 cm、800 rpm、离心5 min收集细胞，弃去上清后置于1 mL匀浆器中，加入1 mL Trizol试剂后研磨（冰盒上操作）。进行RT-PCR分析。采用Trizol（Takara公司）试剂说明书介绍的方法提取组织总RNA，紫外分光光度计测定RNA含量，以总RNA为模板、Oligo（dT）为引物, 应用RT-PCR两步法试剂盒（TaKaRa公司）将mRNA逆转录成cDNA，反应参数：30 ℃、10 min→2 ℃、30 min→99 ℃、5 min→5 ℃、5 min。所得cDNA -20 ℃保存。以反转录所得cDNA为模板，相关引物序列见表 1。上游引物0.5 μL、下游引物0.5 μL，40 μL体系。扩增参数：94 ℃、2 min→（94 ℃、30 s→55 ℃、30 s→72 ℃、30 s）×45循环→72 ℃、5 min。取PCR产物经琼脂糖电泳后用FR200图像分析系统分析。

**1:** 引物序列 Primer sequences

Gene name	Primer sequences（5′-3′)	Product length (bp)	Temperature (℃)
*β-actin*	Upper primer: AGAGG GAAAT CGTGC GTGAC; Under primer: ACATC TGCTG GAAGG TGGAC	224	55
*AQP1*	Upper primer: GGCCACGACCCTCTTTGTCTTCAT; Under primer: TCCCACAGCCAGTGTAGTCGATAG	515	55
*KDR*	Upper primer: GCTCAAGACAGGAAGACCAAGAA; Under primer: TTCCCCAATACTTGTCGTCTGAT	408	55

#### 1.2.6 Western blot检测KDR、AQP1蛋白表达

在RT-PCR提取后剩余物中，加入适量裂解液及蛋白酶抑制剂，4 ℃、以1, 500 rpm离心30 min，取上清液。用考马斯亮蓝法测定蛋白含量后。将5%浓缩胶40 V衡压1 h，10%分离胶60 V恒压3.5 h，湿转14 V恒压14 h，37 ℃摇床封闭2 h，洗膜10 min，3次，转印后加入一抗：KDR多克隆抗体（1:1, 000）或AQP1多克隆抗体4 ℃过夜（1:1, 000）；抗鼠β-actin（1:5, 000）4 ℃过夜。次日加入二抗山羊抗兔抗体（1:700），37 ℃摇床孵育1.5 h，TTBS洗3次×5 min，TBS洗3次×5 min。将硝酸纤维素膜用发光液（Pierce，A、B各100 μL）充分润湿后作用5 min，保鲜膜覆盖，置暗盒中曝光5 min。显影、水洗、定影后观察结果，Quantity one图像分析。目的条带灰度值除以β-actin灰度值进行结果分析。

### 1.3 统计学处理

采用SPSS 13.0统计软件进行分析，计量资料以Mean±SD表示，两组间比较用*t*检验，多组间比较用方差分析，协方差校正，*P* < 0.05为差异具有统计学意义。

## 2 结果

### 2.1 细胞形态学观察

肺腺癌A549细胞在光学显微镜观察，可见细胞生长旺盛，分布密集，大多呈多角形，细胞走向趋于一致，多角形细胞的胞体饱满、有2个-3个扁平而长的突起，细胞核较大，呈卵圆形且多居中（图 1）。

**1:**
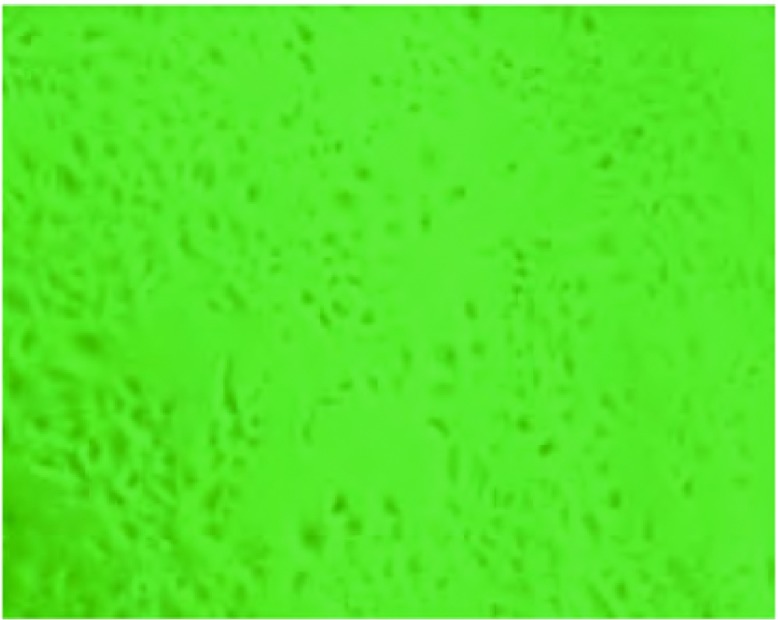
A549细胞光学显微镜下形态（×200） A549 morphology under the light microscope (×200)

### 2.2 各组A549细胞经处理后的OD_490_值

从表 2可以看出：Cetuximab单药处理组抑制率为（30.6±1.2）%；Celecoxib单药处理组抑制率为（29.8±1.4）%；而Cetuximab 1 nmol/L+Celecoxib 25 μmol/L组两药联用的效果，抑制率为（61.4±2.2）%。说明两者联合作用具有协同效应。

**2:** 各组A549细胞OD_490_值（Mean±SD, *n*=9） OD_490_ value after Cetuximab and Celecoxib inhibit A549 (Mean±SD, *n*=9)

Group	OD_490_	Inhibition ratio (%)
Control	0.57±0.06	-
Cetuximab	0.38±0.08^*△^	30.6±1.2^*△^
Celecoxib	0.39±0.05^*△^	29.8±1.4^*△^
Combination	0.27±0.06	61.4±2.2
Compared with control group, ^*^P < 0.05; Compared with Combination group, ^△^*P* < 0.05.		

### 2.3 流式细胞术测定细胞周期分布

如表 3所示，Cetuximab组、Celecoxib组和Combination组G_1_期细胞明显多于对照组，Combination组明显多于Cetuximab组和Celecoxib组，而S期细胞相应减少，差异有统计学意义（*P* < 0.05），M期细胞无明显变化。表明Cetuximab 1 nmol/L+Celecoxib 25 μmol/L组两药联用的效果优于单独用药。

**3:** 对各组549细胞周期的影响（Mean±SD, *n*=3） Cetuximab and Celecoxib effect on A549 cell cycle (Mean±SD, *n*=3)

Group	G_0_/G_1_ phase	S phase	G_2_/M phase
Control	40.9±1.3	48.2±2.4	8.2±0.8
Cetuximab	58.1±3.5^*△^	35.4±2.1^*△^	7.9±1.3
Celecoxib	58.4±2.7^*△^	35.2±2.3^*△^	7.8±2.0
Combination	68.8±4.2^*^	22.35±1.2^*^	10.0±1.6
Compared with control group, ^*^P < 0.05; Compared with Combination group, ^△^*P* < 0.05.			

### 2.4 A549细胞体外侵袭力的变化

如图 2所示，对照组A549细胞穿过滤膜的细胞数量较多，Cetuximab组、Celecoxib组穿膜细胞数明显减少，Combination组穿膜细胞数最少。实验结果表明Cetuximab 1 nmol/L+Celecoxib 25 μmol/L组两药联用能更有效地降低A549细胞的侵袭力。

**2:**
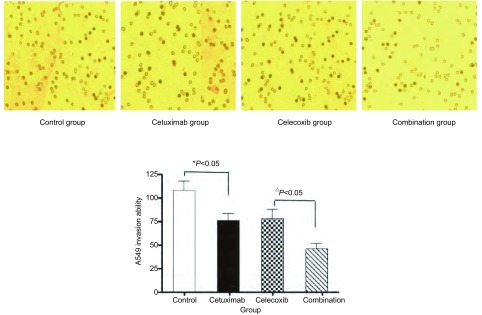
不同处理组处理后A549细胞侵袭能力的影响。和对照组比较，^*^P < 0.05；与Combination组比较，^△^*P* < 0.05。 Changes of invasion ability of A549 cells in each group cells. Compared with control group, ^*^P < 0.05; Compared with Combination group, ^△^*P* < 0.05.

### 2.5 AQP1、KDR mRNA表达

经过相关处理后，AQP1 mRNA、KDR mRNA在Combination组表达量较Cetuximab组、Celecoxib组降低，差异有统计学意义（*P* < 0.05）。较对照组明显降低，差异有统计学意义（*P* < 0.05）（图 3）。

**3:**
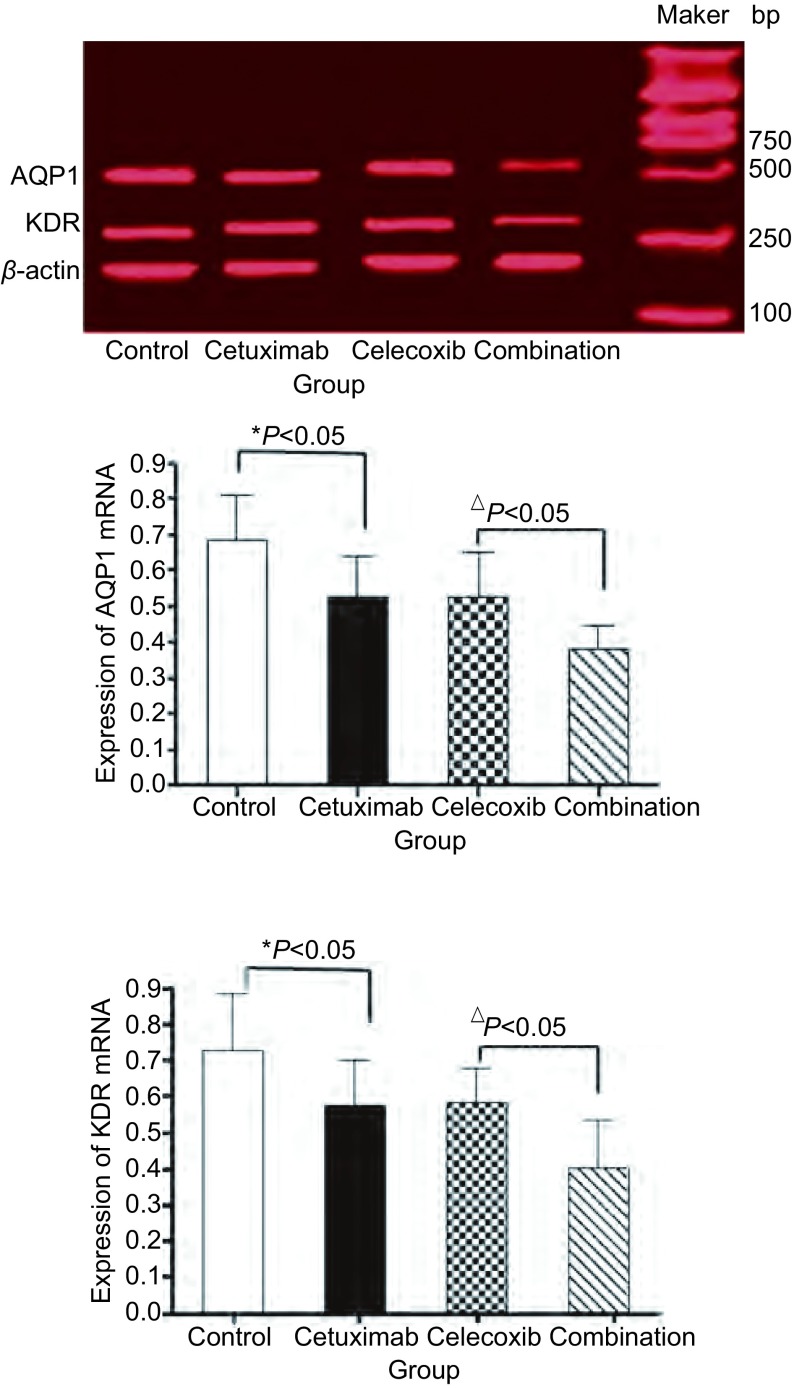
各组细胞AQP1 mRNA、KDR mRNA表达变化。与对照组比较，^*^P < 0.05；与Combination组比较，^△^*P* < 0.05。 Changes of AQP1 and KDR mRNA levels in each group cells. Compared with control group, ^*^P < 0.05; Compared with Combination group, ^△^*P* < 0.05.

### 2.6 AQP1、KDR蛋白表达

给予相关处理后各组AQP1、KDR蛋白表达表达如下（图 4），经过相关处理后，AQP1、KDR蛋白在Combination组表达量较Cetuximab组、Celecoxib组降低，差异有统计学意义（*P* < 0.05）。较对照组明显降低，差异有统计学意义（*P* < 0.05）。

**4:**
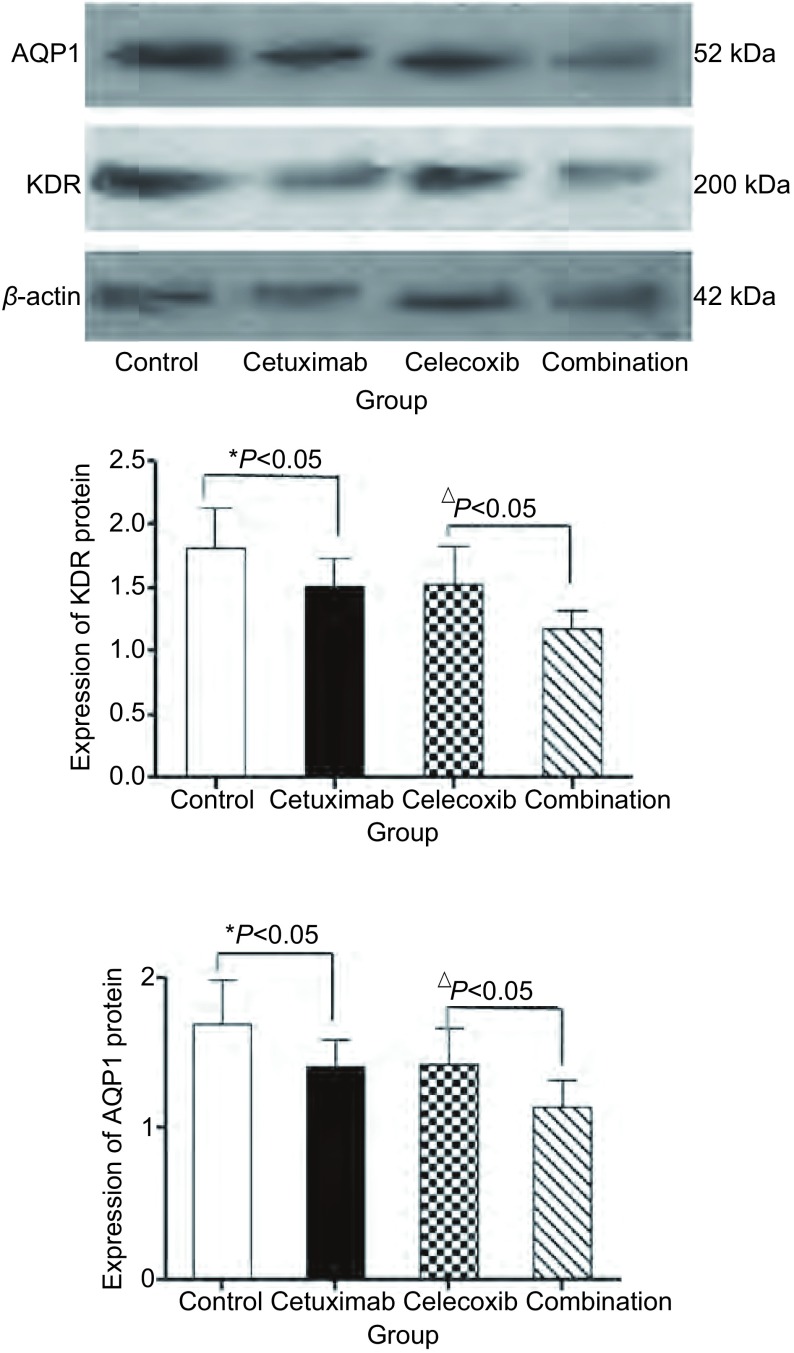
各组细胞AQP1蛋白、KDR蛋白表达变化。与对照组比较，^*^P < 0.05；与Combination组比较，^△^*P* < 0.05。 Changes of AQP1 and KDR protein levels in each group cells. Compared with control group, ^*^P < 0.05; Compared with Combination group, ^△^*P* < 0.05.

## 3 讨论

肺癌是一种临床常见的肺部恶性肿瘤，其死亡率己占癌症死亡率之首，可分为小细胞肺癌和非小细胞肺癌，其中非小细胞肺癌占了到肺癌的85%以上。肺癌的侵袭和转移是肺癌患者治疗失败和死亡的主要原因。而肿瘤血管的生成在肿瘤的浸润和转移过程中起着至关重要的作用^[9]^。因为肿瘤的持续生长必须依赖新生血管生成，大量的新生血管形成是体积 > 2 mm的肿瘤继续生长不可缺少的因素，没有足够的新生血管提供充足的养分，肿瘤的生长潜能将严格受到限制^[10, 11]^。

近年来的研究^[12, 13]^表明KDR及AQP1与肿瘤血管生成关系密切，不仅在血管内皮细胞表达KDR及AQP1，而且肿瘤细胞亦能表达。其中AQP1是肿瘤血管生成的一种新的促进因子，被认为是血管增生的一种标记物。目前大量研究表明许多肿瘤组织、癌细胞系及微血管内皮细胞中AQP1呈高度表达^[14, 15]^，并且其表达量与肿瘤恶性程度有直接关系，恶性程度越高，AQP1表达量越高^[16]^，提示应用水通道蛋白（aquaporins, AQP）的抑制剂或抗AQP抗体降低AQP1的表达将会是新的治疗肺腺癌手段。KDR也是肿瘤血管生成过程中主要的调控因子之一^[17]^。肿瘤细胞分泌血管内皮生长因子（vascular endothelial growth factor, VEGF），除了以旁分泌的形式作用于血管内皮上的KDR，促进血管形成外，还以自分泌的方式作用于自身细胞上的KDR，直接促进肿瘤细胞生长。另外研究发现阻断KDR表达可诱导肿瘤细胞凋亡。因此，抑制KDR表达，不仅可抑制肿瘤血管生成，而且也可阻止肿瘤细胞的生长从而阻断肺癌的生长和转移。故以KDR为靶点抑制血管生成进而控制肿瘤生长，对肿瘤治疗和防止肿瘤远处转移也有重要意义。

近年来研究^[18]^表明，COX-2抑制剂塞来昔布对多种肿瘤细胞有抑制增殖、诱导分化、促进凋亡作用；西妥昔单抗是一种肿瘤分子靶向药物，也广泛应用于基础和临床研究^[19, 20]^。本实验将两者联合应用于A549细胞的体外培养，探讨两者对A549细胞KDR及AQP1表达的影响及对肿瘤细胞的抑制效果。研究发现在A549细胞系的体外作用中，西妥昔单抗和塞来昔布都具有一定的抑瘤效应，联合应用时，二者具有明显的协同效应，可进一步抑制细胞的生长和迁移。西妥昔单抗和塞来昔布单药作用于A549细胞能够明显降低*AQP1*和*KDR*基因和蛋白的表达水平，两药联合时降低*AQP1*和*KDR*基因和蛋白表达的作用更加明显。提示降低*AQP1* 和*KDR*基因和蛋白的表达可能是两者协同作用的分子机制之一。具体机制本研究分析认为与两种药物分别通过抑制COX-2及抗肿瘤作用改善微环境，进而使*AQP1*和*KDR*基因和蛋白表达降低。西妥昔单抗和塞来昔布联合作用后细胞凋亡率更高（*P* < 0.01）；联合用药与单独用药相比，可使细胞发生明显的G_1_期阻滞（*P* < 0.01），细胞侵袭力明显降低。本研究显示将西妥昔单抗和塞来昔布联合应用于肺腺癌的治疗效果更佳，能够为临床提供有益的思路。
